# Innovative Titanium Implants Coated with miR-21-Loaded Nanoparticle for Peri-Implantitis Prevention

**DOI:** 10.3390/pharmaceutics18010142

**Published:** 2026-01-22

**Authors:** Anna Valentino, Raffaele Conte, Pierfrancesco Cerruti, Roberta Condò, Gianfranco Peluso, Anna Calarco

**Affiliations:** 1 Research Institute on Terrestrial Ecosystems (IRET), National Research Council (CNR), Via Pietro Castellino 111, 80131 Naples, Italy; anna.valentino@cnr.it (A.V.); gianfranco.peluso@unicamillus.org (G.P.); anna.calarco@cnr.it (A.C.); 2 National Biodiversity Future Center (NBFC), 90133 Palermo, Italy; 3 Faculty of Medicine and Surgery, Saint Camillus International University of Health Sciences, Via di Sant’Alessandro 8, 00131 Rome, Italy; 4 Institute of Polymers, Composites and Biomaterials (IPCB), National Research Council (CNR), Via Campi Flegrei 34, Pozzuoli, 80078 Naples, Italy; cerruti@unina.it; 5 Department of Clinical Sciences and Translational Medicine, University of Rome “Tor Vergata”, Via Montpellier 1, 00133 Rome, Italy

**Keywords:** microRNA-eluting implant, peri-implantitis, nanoparticles, regeneration, osteointegration

## Abstract

**Background/Objectives:** Peri-implantitis is a chronic inflammatory condition affecting tissues surrounding dental implants and is characterized by progressive marginal bone loss that can ultimately lead to implant failure. Reduced vascularization and impaired immune clearance in peri-implant tissues contribute to persistent inflammation and limited therapeutic efficacy. MicroRNAs (miRNAs), particularly miR-21, have emerged as key regulators of inflammatory responses and bone remodeling. The objective of this study was to develop a bioactive dental implant coating capable of locally delivering miR-21 to modulate inflammation and promote peri-implant tissue regeneration, thereby preventing peri-implantitis. **Methods:** Cationic nanoparticles were synthesized using lecithin and low-molecular-weight polyethylenimine (PEI) as a non-viral delivery system for miR-21. Lecithin was employed to enhance biocompatibility, while PEI functionalization provided a positive surface charge to improve miRNA complexation and cellular uptake. The resulting lecithin–PEI nanoparticles (LEC–PEI NPs) were incorporated into a chitosan-based coating and applied to titanium implant surfaces to obtain a sustained miR-21–releasing system (miR21-implant). Transfection efficiency and biological activity were evaluated in human periodontal ligament fibroblasts (hPDLFs) and compared with a commercial transfection reagent (Lipofectamine). Release kinetics and long-term activity of miR-21 from the coating were also assessed. **Results:** MiR-21-loaded LEC–PEI nanoparticles demonstrated significantly higher transfection efficiency than Lipofectamine and retained marked biological activity in hPDLFs relevant to peri-implantitis prevention. The chitosan-based nanoparticle coating enabled controlled and sustained miR-21 release over time, supporting prolonged modulation of inflammatory and osteogenic signaling pathways involved in peri-implant tissue homeostasis. **Conclusions:** The miR21-implant system, based on lecithin–PEI nanoparticles incorporated into a chitosan coating, represents a promising therapeutic strategy for peri-implantitis prevention. By enabling sustained local delivery of miR-21, this approach has the potential to preserve peri-implant bone architecture, modulate chronic inflammation, and enhance the osseointegration of titanium dental implants.

## 1. Introduction

Peri-implantitis (PI) is an inflammatory disease characterized by progressive bone destruction around dental implants. The condition leads to the loss of supporting bone tissue, thereby compromising the stability of the implant–bone interface. Consequently, peri-implantitis is recognized as one of the main causes of implant failure and has received increasing scientific attention [1,2,3,4]. Compared to natural periodontal tissues, peri-implant tissues exhibit fewer blood vessels between the marginal bone and the junctional epithelium. This reduced vascularization limits immune-cell infiltration and the clearance of microbial pathogens or titanium debris released from implant surfaces [5,6,7]. As a result, peri-implant infections tend to progress more rapidly and persistently, leading to severe local inflammation, immune dysregulation, and marginal bone resorption [8].

Although dental implants generally show high success and survival rates, PI remains a major clinical challenge due to its complex multifactorial etiology and the limited efficacy of available therapeutic strategies. Therefore, understanding the molecular mechanisms underlying peri-implant inflammation and bone loss is essential for developing innovative preventive and therapeutic approaches [9,10,11]. MiRNAs are a group of short non-coding RNA molecules that play a crucial role in post-transcriptional control of gene expression, particularly in biological processes such as inflammation, fibrosis, and bone remodeling [12,13,14]. Among them, miR-21 plays a crucial role in modulating immune responses and promoting osteogenic differentiation [15,16,17]. Dysregulation of miR-21 has been associated with various inflammatory and degenerative diseases, including periodontitis and peri-implantitis, suggesting its potential as a therapeutic target for maintaining peri-implant tissue homeostasis [18,19]. In this context, the delivery of miR-21 to peri-implant cells represents a promising strategy to modulate local inflammation and stimulate tissue regeneration. However, the therapeutic application of miRNAs is limited by their instability and poor cellular uptake. To overcome these challenges, cationic nanoparticles based on lecithin and low-molecular-weight polyethylenimine (PEI) were synthesized as non-viral vectors for miR-21 delivery. Lecithin, a natural phosphatidylcholine of plant origin, is biocompatible and widely used in the formulation of nanosized drug delivery systems [20,21,22,23]. Nevertheless, its neutral surface charge hampers effective binding of the negatively charged miRNAs. Functionalization of lecithin nanoparticles with PEI provides a positive surface potential, enabling strong electrostatic interaction with miR-21 and enhancing intracellular delivery while reducing PEI cytotoxicity through stable surface immobilization [24]. The resulting lecithin–PEI nanoparticles (LEC-PEI NPs) were incorporated into a chitosan-based coating applied onto titanium implant surfaces. Chitosan, a biocompatible and antimicrobial polysaccharide [25,26], was selected for its ability to form stable coatings capable of sustained nanoparticle release [27,28]. The miR-21-loaded chitosan coating, thus, provides a bioactive interface capable of promoting peri-implant cell regeneration while preventing bacterial colonization and inflammatory bone resorption. This study aims to develop and characterize a miR-21 nanoparticle-eluting dental implant designed to prevent peri-implantitis by locally modulating inflammatory pathways and enhancing osteogenic activity at the implant–tissue interface.

## 2. Results and Discussion

### 2.1. Preparation of Cationic Lecithin–PEI Nanoparticles (LEC-PEI NPs)

The latest scientific evidence supports a critical role of miRNAs in osteointegration. In comparison to simple drug-eluting implant treatments, polinucleotide-based therapies offer the advantage of simultaneously regulating various cellular targets [29,30]. However, the weak miRNA pharmacokinetic profile limits the clinical applications. These constraints could be addressed by the use of cationic polymeric vectors which act as a shield against enzyme breakdown, improving nucleic acid bioavailability. Polyethyleneimine (PEI), characterized by a strong cationic charge density, is known to activate an endocytic mechanism that causes fast intracellular uptake and the release of polynucleotides (the “proton sponge effect”), representing an ideal candidate for gene delivery [31]. Moreover, the severe cytotoxicity caused by the high positive surface charge of PEI’s [32] can be avoided through bounding with nanoparticle vectors.

Based on these considerations, in the present study was described a two-step procedure for the preparation of LEC-PEI NPs in which lecithin NPs were firstly synthesized by a nanoemulsion method, followed by PEI surface functionalization. The optimization of the synthethic procedure was achieved through the determination of the optimal process parameters for the nanoemulsion, in order to obtain NPs with optimized size and polydispersity index (PDI). In detail, a range of lipophilic/hydrophilic ratios were investigated, and the influence of sonication time and amplitude was also studied. As reported in Table S1, sonication time and amplitude had minimum influence on the nanoparticles sizes, while the volume ratio of O/W emulsion was directly proportional to nanoparticles size. Nanoparticles obtained with a O/W ratio of 1:4 (LEC-NPs) showed a stable hydrodynamic diameter of 103.9 ± 11.2 nm. This preparation was then selected for the subsequent surface modification reaction in which PEI was grafted to lecithin to impart a cationic surface charge to the negatively charged phospholipid nanoparticles, as reported by Conte et al. [12].

**Figure 1:**
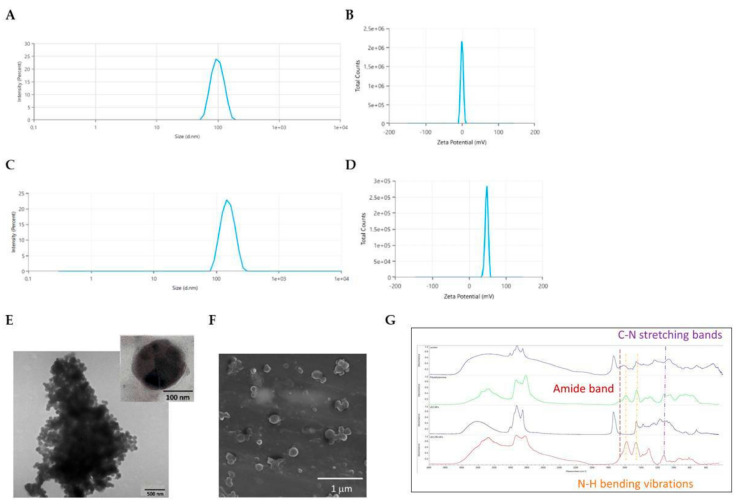
Physico-chemical characterization of LEC NPs and LEC-PEI NPs. Hydrodynamic diameters (**A**) and zeta potential (**B**) of LEC NPs; hydrodynamic diameters (**C**) and zeta potential (**D**) of LEC-PEI NPs. (**E**) TEM and (**F**) SEM images of LEC-PEI NPs showing the morphology, size, and uniformity of the nanoparticles. (**G**) FT-IR spectra.

The grafting of PEI on a polymer surface is usually achieved by aminolysis of polymer–ester bonds, which is an easily performed chemical modification allowing free -NH_2_ groups to be incorporated along the lipophilic chain [33]. The amino groups of branched PEI react with lecithin esters, forming amide bonds. In this way, the net cationic charge of lecithin NPs increases and the amine groups of PEI, in stoichiometric excess, are available for nucleic acid complex formation. Aminolysis was employed to maintain structural integrity of the lecitin nanoparticles, promoting selective covalent grafting and circumventing solvent-induced phospholipid degradation observed in solution-phase attempts. LEC-NPs were resuspended in an isopropanol–polyethylenimine solution at different concentrations (12%, 24%, and 48% *w*/*v*) for periodic intervals of up to 60 min. According to a quantitative ninhydrin assay, the LEC-NPs surface contained the most -NH_2_ groups (43 mmol -NH_2_/g) after being treated with 24% PEI for 15 min at 50 °C. DLS analysis showed a small increase in hydrodynamic diameter (145.1 ± 23.6 nm) (Figure 1A,C), while the zeta potential ehanced drastically, from 0.3 ± 0.02 to 47.7 ± 0.9 mV (Figure 1B,D), supporting the effective surface alteration. TEM and SEM were used to further describe the NPs’ size and shape, higlighting a smooth surface with a regular spherical shape (Figure 1E,F). Moreover, such determinations were repeated at regular intervals up to 90 days of storage. Both LEC-NPs and LEC-PEI NPs were stable with optimal re-dispersion abilities in water.

Chemical characterization of the NPs was performed by FTIR spectroscopy (Figure 1G). This tecnhique was used to compare LEC-NPs and LEC-PEI NPs in order to evaluate the occurrence of interactions between lecithin and polyethyleneimine during nanoparticle formation. In the LEC-NPs, the spectrum largely resembles that of pristine lecithin, indicating that nanoparticle formation alone does not significantly alter the chemical structure of the phospholipid. In particular, the ester C=O band at ~1735 cm^−1^ and the phosphodiester bands (1240–1220 cm^−1^) remain unchanged, confirming the absence of new chemical interactions in LEC-NPs. Conversely, the LEC-PEI NPs spectrum exhibits clear spectral modifications compared to LEC-NPs, indicating successful interaction between PEI and lecithin. Notably, new bands appear in the 1600–1550 cm^−1^ region, which are absent or very weak in LEC-NPs and can be attributed to N–H bending vibrations of PEI. Similarly, the appearance of C–N stretching bands in the 1200–1100 cm^−1^ region not present in LEC-NPs further confirms the successful association of PEI with the lecithin matrix, suggesting its incorporation into the nanoparticle structure. Moreover, no amide I/II band (1650 cm^−1^) distinctive of pure PEI are detected in LEC-PEI NPs, which confirms amide formation due to ester aminolysis (Figure 1G).

PEI transfection is subject to apoptotic and necrotic effects, and is added to a moderate genotoxic influence [34], due to the elevated polymeric molecular weight [35,36]. Then, the use of low-molecular-weight PEI for nanoparticles functionalization should reduce cytotoxicity [37]. As confirmed, biocompatibility was assayed in hPDLF after incubation in media that had different concentrations of NPs (5, 50, 100, 250, and 500 µg/mL) for 72 h. As reported in Figure S1A, no differencies in cell viability were observed for the tested concentrations (>90% cell viability). This agrees with the LDH assay which showed that LEC-PEI NPs did not induce LDH release outside the cell, maintaining cell-membrane permeability/integrity (Figure S1B).

### 2.2. Physical Properties of LEC-PEI NPs/miRNA Complexes (miR-NPx)

**Figure 2:**
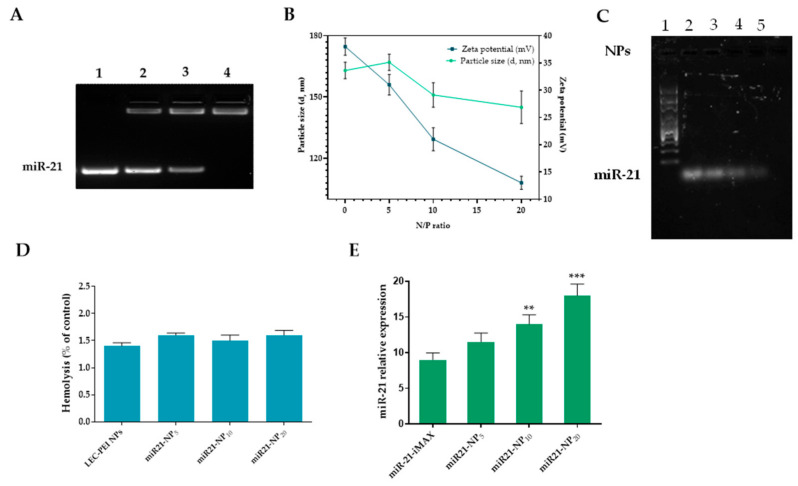
Nanocomplex (miR21-NP_x_) characterization. (**A**) Agarose retardation assay of miR21-NP_x_ in function of N/P ratio. Lane 1: free miR-21; Lane 2: miR21-NP_5_; Lane 3: miR21-NP_10_; Lane 4: miR21-NP_20_. Following gel electrophoresis, band intensities were measured using ImageJ software v.1.54 and plotted over time, with free miR-21 serving as the control. (**B**) Hydrodynamic diameter and zeta potential of miR21-NP_x_ at various N/P ratios (mean ± SD, *n* = 6). (**C**) FBS protection assay for miR21-NP_x_ after incubation in 10% FBS for 30 min. Nuclease activity in the serum was inactivated by heating the nanoparticles at 65 °C for 20 min. Lane 1: RNA ladder 1 Kb; Lane 2: free miR21; Lane 3: miR21-NP_5_; Lane 4: miR21-NP_10_; Lane 5: miR21-NP_20_. Free miR-21 was used as control. Images are representative of three independent experiments. (**D**). Hemolysis of red blood cells after incubation with water (+), 1 × PBS (−), miR21-NP_5_, miR21-NP_10_, and miR21-NP_20_ for 2 h at 37 °C. (**E**). miR21-NP-transfection efficiency respect to miR-21-iMAX. The bars represent the mean ± SD, *n* = 3. Statistically significant variations: ** *p* < 0.01, and *** *p* < 0.001.

MiRNAs are short non-coding RNAs acting as post-transcriptional repressors of gene expression [38]. Given the presence of cellular RNase is able to rapidly degrading small nucleic acids, the prerequisite of an efficient miRNA delivery system is that such a vector must preserve nucleic acids from the degradation activity of nucleases [39]. The complexation behavior of LEC-PEI NPs with 50 pM of miR-21 was studied by agarose gel electrophoresis at different N/P proportions (5:1, 10:1, 20:1 miR21-NP_5_, miR21-NP_10_, and miR21-NP_20_, respectively). The intensity of the band corresponding to free miRNA decreased with a rise in the N/P ratio (Figure 2A). Specifically, LEC-PEI nanoparticles achieve full miRNA complexation at ratios exceeding 10:1. The formation of these nanocomplexes is associated with a progressive reduction in particle size, as the N/P ratio increases from 1:1 to 20:1 while maintaining unchanged colloidal stability. On the other side, a statistically significant decrease in ζ-potential value was evident, showing a shift from ≈38 mV of naked LEC-PEI NPs to ≈21 mV (*p* < 0.05) and 13 mV (*p* < 0.001) with the higher N/P ratio (miR21-NP_10_ and miR21-NP_20_, respectively) (Figure 2B). Moreover, the residual net-positive charge of nanocomplex favors the electrostatic interactions necessary to bind with cell membranes and permeate into the cytoplasm via endosomal transport [40,41].

To accurately simulate physiological conditions, the stability of the nanocomplexes and their nuclease-protective abilities were assessed by gel mobility assays at different N/P ratios in the presence of serum (Figure 2C). The results showed that ~90% of unchanged miRNA was preserved when complexed with miR21-NP_10_ and miR21-NP_20_ after 24 h of incubation, while the free miRNA was degraded after 1 h. Such data were confirmed by the size and zeta potential measurements. miR21-NP_10_ showed a particle size of 124 nm with a zeta potential of 23 mV corresponding to a variation of 2 nm and 0 mV from the initial values of 122 nm and 23 mV. Similarly, miR21-NP_20_ exhibited a particle size of 105 nm with a zeta potential of 14 mV, showing a variation of 2 nm and 1 mV from the initial 103 nm and 12 mV values. Blood compatibility of nanocomplexes was assessed by haemolitic assay as described in ISO 10993–4:2017 (Biological evaluation of medical devices—Part 4: Selection of tests for interactions with blood; Provides general requirements for evaluating the interactions of medical devices with blood). As reported in Figure 2D, all samples showed a haemolysis rate less than 5% after 60 min of blood-cell contact. These results suggest that LEC-PEI NPs can protect nucleic acids from nucleases for long periods of time and that they are stable under in vivo conditions [42,43,44].

The transfection efficiency of miR21-NPx (x = 5:1, 10:1, 20:1) was assessed by real-time PCR with Lipofectamine RNAiMAX (iMAX) used as control. MiR21 nanocomplex at N/P ratio 10:1 leads to a considerable (*p* < 0.01) 1.5-fold increase in Cy5-positive hPDLF cells whereas the N/P ratio 20:1 had a 2-fold increment (Figure 2E). Additionally, the transfection with systems having N/P ratios lower than 20 had a transferable transfection efficiency because of the fragile interactions between NPs and miRNA. Based on these data, miR21-NP_20_ was selected for subsequent investigation. Biocompatibility of miR21-NPs was further confirmed using CCK-8 and LDH assay (Figure S1C,D).

### 2.3. Preparation and Characterization of Chitosan Doped with miR21-NP_20_ (miR21-NPs)

**Figure 3:**
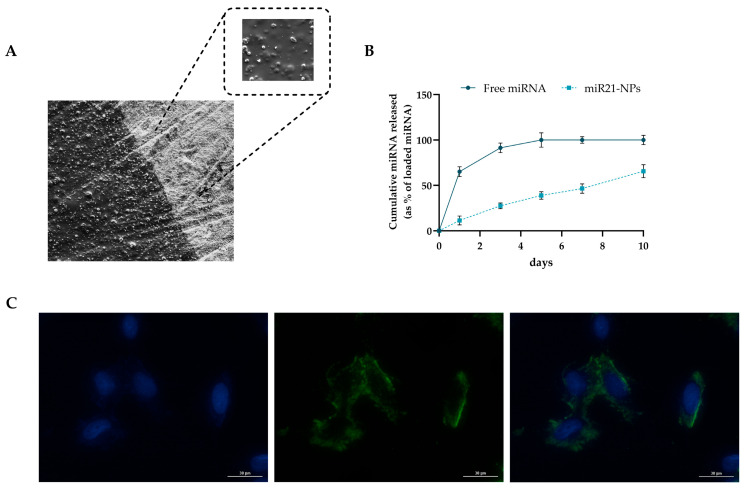
Characterization of miR21-NPs. (**A**). Scanning electron microscopy (SEM) photograph (left: low magnification, scale bar = 1 μm; right: high magnification, scale bar = 100 nm) of miR21-implant. (**B**). Cumulative release of fluorescent miRNA from miRNA-stent after 10 days of incubation (*n* = 8 each). The percentage of incremental quantities of released miRNA was plotted against time. (**C**). Fluorescent microscopy analysis of cellular uptake of mi21-NPs released after 24 h. Transfected cells are green, nuclei are stained in blue. Scale bars, 30 μm.

Gene-eluting implants represent a promising strategy to deliver effective local concentrations of nucleic acids, such as siRNAs or miRNAs, while minimizing systemic distribution and off-target effects [45]. To date, several therapeutic genes have been explored to modulate tissue regeneration and prevent pathological remodeling [46,47]. However, implants are generally recognized as “foreign objects” by the host’s immune system, which can trigger inflammation and compromise integration. Highly biocompatible coatings, such as chitosan (CHI), can camouflage the implant surface, attenuating foreign body responses and modulating the release kinetics of loaded bioactive agents [48]. Additionally, CHI coatings possess intrinsic antibacterial properties, counteracting pathogens commonly associated with peri-implantitis, including *Porphyromonas gingivalis*, *Tannerella forsythia*, *Aggregatibacter actinomycetemcomitans*, *Prevotella intermedia*, and *Treponema denticola*, thus reducing the risk of infection [49]. To exploit the synergistic effect of CHI in supporting miRNA delivery, miR21-NP_20_ were embedded into a CHI coating on titanium implants. Scanning electron microscopy revealed a thin and uniform polymer layer on the implant surface, with nanoparticles structurally intact and well-dispersed within CHI (Figure 3A). Similar strategies have been reported in the literature: Qiu et al. functionalized stents with 2-N,6-O-sulfated chitosan (26SCS), demonstrating that surface coating can modulate physical and chemical properties without altering mechanical integrity [50], while Wang et al. applied a layer-by-layer approach to fabricate biocompatible chitosan/hyaluronic acid nanoparticles for miR-21 delivery, enhancing proliferation and osteogenesis in human bone marrow mesenchymal stem cells [51]. The release profile of miR-21 from CHI-coated implants was evaluated using fluorescently labeled miRNA (MISSION^®^ miRNA, miRNA-NP_20_) and FACS analysis. Nanoparticles embedded in CHI exhibited an initial burst release of ~10% during the first hours, followed by sustained release, reaching 46.11 ± 3.29% after 7 days and 65.64 ± 5.28% at 10 days. In contrast, free miRNA dispersed in CHI was almost completely released within 3 days, highlighting that nanoparticle encapsulation is essential to prolong the bioavailability and efficacy of the gene-eluting implant (Figure 3B). These results corroborate the hypothesis that chitosan-based coatings can provide a controlled, sustained release of miRNAs while promoting cell proliferation, osteogenic differentiation, and angiogenesis, thereby enhancing peri-implant tissue regeneration and preventing peri-implantitis.

hPDLF cells cultured directly on the miRNA-implant had high and stable intracellular delivery of fluorecent miRNA after 24 h of incubation (Figure 3C) confirming the coating’s ability to release nanoparticles, allowing them to be internalized by cells. Moreover, the safety of the miR21-implant was assessed using CCK-8 and LDH assay (Figure S1E,F).

### 2.4. MiR21-NPs Accelerate/Facilitate Wound-Healing Process and Induce Angiogenesis on hPDLF

The restoration of peri-implant tissues is a complex biological process involving multiple cellular interactions and dynamic remodeling of the extracellular matrix to achieve structural and functional recovery [52]. Several interconnected mechanisms regulate cellular behavior during peri-implant healing, including inflammation control, extracellular matrix reorganization, epithelial regeneration, fibroblast proliferation and migration, and the formation of new blood vessels [53].

**Figure 4:**
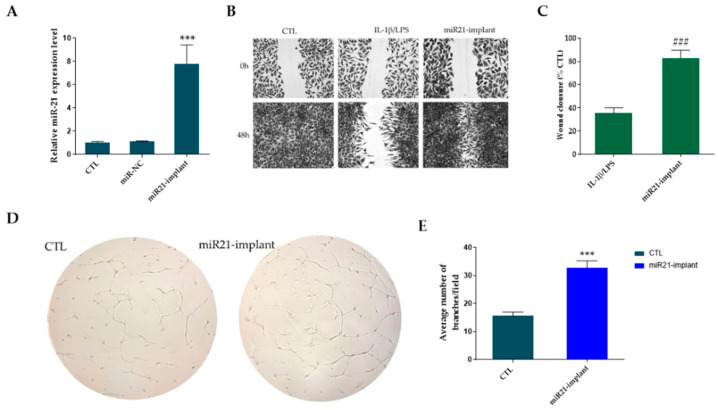
Influence of forced expression of miR-21 on proliferation, migration, and new angiogenesis. Cells were cultured for 48 h after transfection with miR-NC and miR21-implant. The untreated cells were used as control (CTL). (**A**) Tranfection efficiency of miR-21-NPs released by implant in respect to miR-NC. (**B**,**C**). Representative images of wound closure at 0 and 48 h following treatment with IL-1β/LPS alone or in combination with miR21-implant, together with the corresponding quantitative analysis, are shown. Scale bars correspond to 50 µm (*n* = 3). (**D**) Representative optical images of HUVEC tube formation after 4 h of incubation with CTL (positive control treated with VEGF at 20 ng/mL) or miR21-implant; images were acquired at 20× magnification. (**E**) Quantitative analysis of tube formation was performed using ImageJ software (version 1.54j) with the Angiogenesis Analyzer plugin. Statistical significance is indicated as ### *p* < 0.001 for miR21-implant versus IL-1β/LPS and *** *p* < 0.001 for miR21-implant versus CTL. Results are reported as the mean ± SD of three independent experiments (*n* = 3).

In our study, miR21-NPs transfection released by a titanium implant (miR21-implant) in hPDLFs significantly (*p* < 0.001) increased miR-21 expression in respect to miR-NC group (Figure 4A). To evaluate the impact of miR21 on the migration of periodontal fibroblasts under serum-starved conditions, hPDLFs were incubated with a miR21-implant for 48 h. A wound-healing assay revealed that, under normal conditions, untreated cells migrated to close the wound within 48 h. In contrast, exposure to inflammatory stimuli IL-1β/LPS inhibited migration toward the wound center, resulting in markedly reduced wound closure. Notably, hPDLFs treated with a miR21-implant but previously treated with LPS/IL-1b exhibited faster gap closure compared to cells treated only with IL-1β/LPS (Figure 4B). Quantitative analysis of the wound-healing assay confirmed that miR21-implant significantly promoted hPDLF migration, achieving approximately 78% wound closure versus 36% in the IL-1β/LPS group (Figure 4C). These results indicate that miR-21 stimulates regenerative cellular pathways involved in peri-implant healing, supporting its therapeutic potential in the prevention of peri-implantitis through enhanced osteogenic and angiogenic responses. Among these, angiogenesis is particularly crucial, as it ensures adequate oxygen and nutrient supply to the regenerating peri-implant tissue. However, in the case of peri-implantitis, a chronic inflammatory disease characterized by bacterial infection, tissue degradation, and bone resorption, angiogenic activity can become disrupted or insufficient, thereby compromising healing and promoting disease progression [54]. To investigate angiogenic potential, the tube formation assay is frequently employed as an in vitro model to observe the capacity of endothelial cells to align and form capillary-like networks. This method provides a useful platform for studying how biomolecules, growth factors, and biomaterials influence vascularization and tissue regeneration. As shown in Figure 4D, when HUVECs were cultured with miR21-implant-released medium, the number of branches was significantly higher (*p* < 0.001) compared with the VEGF-treated positive control. Quantitative evaluation of HUVEC branching revealed that treatment with the miR21-implant resulted in an increase of approximately 40% relative to the control group (Figure 4E).

### 2.5. MiR-21 Upregulation Modulates Bone Mineralization and Promotes Osteogenic Differentiation

Geng et al. demonstrated that titanium surfaces functionalized with miR-21-loaded nanocapsules promoted osteointegration and mineralization by enhancing osteogenic differentiation and suppressing osteoclastic activity [52].

**Figure 5:**
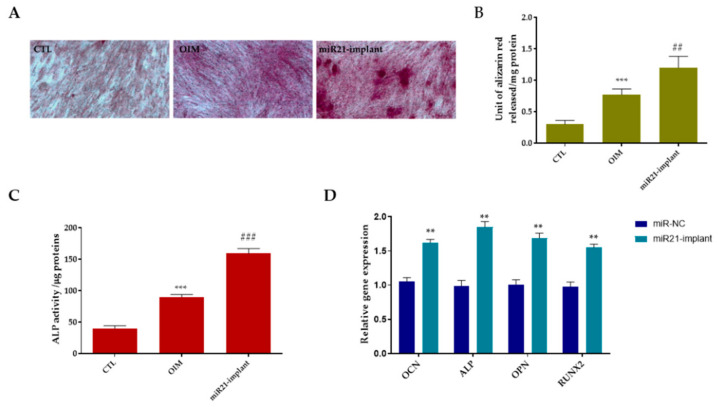
hPDLFs osteogenic gene differentiation and ALP activity analysis. (**A**) Representative transmitted light microscopy images (20× magnification) of Alizarin Red S-stained mineralized nodules formed after 21 days of culture. Cells maintained in osteogenic induction medium (OIM) served as the positive control. (**B**) Quantitative analysis of Alizarin Red S staining. Data are representative of three independent experiments. Statistical significance is indicated as *** *p* < 0.001 versus CTL, ## *p* < 0.01 versus OIM. (**C**) Semiquantitative analysis of ALP activity of cells cultured in presence of miR21-implant until 21 days. The result was representative of three different experiments. *** *p* < 0.001 OIM versus CTL, and ### *p* < 0.001 versus OIM. (**D**) hPDLF osteogenic markers were quantified by quantitative real-time PCR (qPCR) and normalized to Gapdh as a housekeeping gene. (**B**) The expression of the same gene panel was also evaluated following treatment with miR-NC and the miR21-functionalized implant. Relative gene expression levels were calculated using the comparative cycle threshold method (2^−ΔΔCt^). Data are presented as mean ± standard deviation (SD) from three independent experiments (*n* = 3). Statistical significance was defined as ** *p* < 0.01 compared with the miR-NC group.

At the mechanistic level, the delivery of miR-21 stimulated the PI3K/Akt signaling cascade, leading to enhanced expression of osteogenic differentiation markers, including RUNX2 and osteocalcin, leading to improved bone formation and implant anchorage. In vitro, miR-21 enhanced mesenchymal stem-cell proliferation and angiogenic factor secretion, while in vivo it increased bone–implant contact and mechanical fixation strength. In this experimental framework, the osteoinductive potential and underlying molecular effects of miR21-NP_20_ released from the titanium implant were investigated using Alizarin Red staining, alkaline phosphatase (ALP) activity assays, and quantitative real-time PCR (qRT-PCR). As illustrated in Figure 5A, Alizarin Red staining performed on day 21 demonstrated that human periodontal ligament fibroblasts (hPDLFs) exposed to the miR21-functionalized implant formed more extensive and densely mineralized nodules compared with both the osteogenic induction medium (OIM) and control (CTL) groups, indicating enhanced matrix mineralization. These qualitative observations were corroborated by quantitative analysis (Figure 5B). In parallel, ALP activity was markedly elevated in hPDLFs cultured in the presence of the miR21-implant relative to OIM and CTL conditions (Figure 5C). Given that ALP represents a well-established early marker of osteogenic commitment, this increase suggests a pronounced shift in mesenchymal cells toward osteoblastic differentiation. To further elucidate the molecular response induced by miR-21, qRT-PCR was performed to assess the expression of osteogenesis-related genes. After 21 days of culture, hPDLFs in contact with the miR21-implant exhibited a significant upregulation (*p* < 0.01) of all analyzed osteogenic markers compared with the control group (Figure 5D), including alkaline phosphatase (ALP), osteocalcin (OCN), osteopontin (OPN), and RUNX2. Collectively, these findings align with previously reported evidence highlighting the pro-regenerative and anti-inflammatory functions of miR-21 in bone and oral tissue regeneration models. For example, a recent study reported that regulatory T cell-derived exosomes enriched in miR-21 enhanced the osteogenic differentiation of human periodontal ligament stem cells (PDLSCs) by stimulating alkaline phosphatase (ALP), Runx2, and Osterix expression, thereby improving periodontal tissue regeneration in vivo [53]. Furthermore, miR-21 has been shown to exert anti-inflammatory effects in models of periodontitis by downregulating pro-inflammatory cytokines and attenuating bone resorption, indicating its relevance in maintaining peri-implant tissue homeostasis [54]. Other studies have elucidated its mechanistic roles in bone remodeling: miR-21 promotes osteoblast differentiation while modulating osteoclast activity through PTEN/PI3K/Akt/HIF-1α signaling, thus, facilitating osteoblast–osteoclast coupling essential for bone regeneration [55].

Under physiological conditions, periodontal ligament fibroblasts, endothelial cells, and dental pulp stem cells maintain a homeostatic phenotype supporting tissue turnover and repair. However, in response to pathological stimuli such as bacterial biofilm accumulation or oxidative stress, these cells undergo phenotypic alterations characterized by decreased expression of structural and osteogenic markers and increased release of inflammatory cytokines and chemokines [56]. MiR-21 appears to counteract these detrimental effects by restoring proliferative, osteogenic, and pro-angiogenic signaling, thereby preventing peri-implant inflammation and bone loss while promoting stable osseointegration.

All together, these results demonstrate that the miR21-implant represents a promising strategy to prevent peri-implantitis from causing bone loss and inducing osteointegration with an implant that releases miR-21 on hPDLF cells, accelerating proliferation, migration, and differentiation of periodontal cell types.

## 3. Materials and Methods

### 3.1. Materials

Soybean lecithin (Phospholipon 90G, 90% *w*/*w* of phosphatidylcholine) was purchased from Lipoid (Ludwigshafen, Germany). MiRNA-21 mimics a mature miRNA sequence (CAACACCAGUCGAUGGGCUGU), MISSION^®^ miRNA, miRNA negative control (NC-miR): 5′-UUCUCCGAACGUGUCACGUTT-3′, RNAi reporter control and the transfection reagent Lipofectamine RNAiMAX were obtained from Invitrogen (Thermo Fisher, Milan, Italy). Branched polyethylenimine (PEI, 800 da), coconut oil analytical standard, Glycerol ≥ 99.5%, phosphate-buffered saline (PBS), and deacetylated chitosan from crab shells were obtained from Euroclone (Milan, Italy). GelStar was purchased from Roche (Milan, Italy). All other reagents were of analytical grade and used without further purification.

### 3.2. Preparation of PEI-Functionalized Lecithin Nanoparticles (LEC-PEI NPs)

Lecithin nanoparticles (LEC NPs) were prepared by an oil/water (O/W) emulsion method. A determined amount of lecithin was dissolved under magnetic stirring in coconut oil to obtain a final lecithin/oil ratio of 1:10 (*w*/*w*). The lipophilic phase was then added in a different volume (1–10 mL) of highly purified water. Nanoemulsion was produced through cold sonication (8 min at amplitude of 60% in an ice refrigerated container; Digital Sonifier 450, Branson, Italy), leaving the stabilized preparation under magnetic stirring at a room temperature overnight. The formed nanoparticles were collected by centrifugation, (18,000 rpm, 4 °C, 45 min; 5718R, OHAUS, Milan, Italy), washed with double-distilled water, and dried.

Positive surface charge functionalization was obtained by resuspending LEC NPs in an isopropanol–polyethylenimine solution (prepared in different concentrations—12/24% *w*/*v*) for 15 min at 50 °C. The dispersion was then centrifuged (15,000 rpm, 25 °C, 45 min), and nanoparticle pellets were washed with milliQ water and freeze-dried.

### 3.3. Characterization of PEI-Functionalized Lecithin Nanoparticles (LEC-PEI NPs)

#### 3.3.1. Light Scattering and Electron Microscopy

The effect of PEI concentration on the size and zeta potential of LEC-PEI nanoparticles was determined by dynamic light scattering (DLS) and electrophoretic light scattering (ELS) using a MalvernNano ZS (Malvern Instruments Ltd., Malvern, UK). All measurements were carried out at 25 °C using the detection of backscattered light at an angle of 173°. Each analysis consisted of 15 consecutive subruns. Electrophoretic mobility values were converted to zeta potential using the Smoluchowski approximation. Information on the size and morphology of the nanoparticles were collected with a FEI Tecnai G2 Spirit TWIN 120 kV transmission electron microscope (TEM) (Thermo Fisher Scientific, Norristown, PA, USA) and a FEI Quanta 200 FEG scanning electron microscope (SEM) (Thermo Fisher Scientific, Norristown, PA, USA). TEM samples were prepared by deposition of a drop of NP dispersion on a carbon-coated 200-mesh copper specimen grid, while for SEM analysis the samples were deposited on an aluminum stub and left to dry, then coated with AuPd using a sputtering device (MED 020, Bal-Tec AG, Pfäffikon, Switzerland).

#### 3.3.2. FTIR-ATR

FTIR-ATR spectra were recorded on a Perkin Elmer Spectrum 100 spectrophotometer (PerkinElmer, Waltham, MA, USA), equipped with a Universal ATR diamond crystal sampling accessory. Spectral data were collected by averaging 64 scans over the 4000–480 cm^−1^ range, with a spectral resolution of 4 cm^−1^. Before analysis, samples were dried under a vacuum at 60 °C for 24 h.

#### 3.3.3. Coating Stability

PEI coating stability was studied by measurements of in vitro resistance at increasing temperatures and times of storage. LEC-PEI NPs were suspended in water, sealed into glass vials, and stored at 37 °C in the absence of light. The size of the nanoparticles was measured by PCS after 0, 7, 14, 21, 28, 60, and 90 days of storage, setting an automated temperature trend in the range of 17–50 °C.

### 3.4. Preparation and Characterization of LEC-PEI miRNA Complexes (miR-NPs)

To determine the optimal N/P ratio between miRNA and LEC NPs, 50 pM miR-21 was mixed with different concentration of nanoparticles in 150 mM NaCl, pH 7.4 at 37 °C for 30 min to form the miRNA nanocomplexes (miR-NPs). Free miR-21 was used as a control. Dynamic light scattering (DLS) with a Malvern Zetasizer (Malvern Instruments Ltd., Malvern, UK) was used to measure the diameter and zeta potential of the nanocomplexes as reported by Conte et al. [12].

*Gel retardation*. The DNA binding abilities of LEC-PEI nanoparticles were examined by the gel retardation assay as reported by D’Ajala et al. [57]. miR-NPs were electrophoresed on an agarose gel containing 1X that of nucleic acid gel stain (Gel-Star, BMA, Milan, Italy) and with Tris-acetate EDTA (TAE) running buffer at 80 V for 90 min. Nucleic acid bands were visualized using a Gel Doc 2000 imaging system (Bio-Rad, Milan, Italy) equipped with a UV transilluminator. Band densitometry and background subtraction were performed with Quantity One software (version 1.1, Bio-Rad).

*Serum stability*. The stability of miR-NPs complexes was confirmed after the incubation of nanocomplexes in DMEM supplemented with 10% FBS at 37 °C. At a pre-set time (1 to 6 h), the diameter and zeta potential of the miR-NPs complexes were measured as described earlier.

*DNAse I degradation assay*. The DNase protection assay was performed to evaluate the capability of LEC-PEI NPs to protect miR-21 from nuclease degradation. Briefly, miR-NPs were incubated with 10% FBS for 30 min at 37 °C.

### 3.5. Hemolysis Assay

Hemocompatibility of nanocomplexes was determined in accordance with ISO 10993-4 (ISO 10993: Biological evaluation of medical devices—Part 4: Selection of tests for interactions with blood) [58]. Fresh, human whole blood with sodium citrate was collected from AMES Diagnostic Center (Casalnuovo di Napoli, Italy). A total of 2 mL of human blood was centrifuged for 10 min at room temperature at 4500 rpm to separate out plasma, then a settled erythrocyte pellet was re-suspended in an equal volume of normal saline (Sodium Chloride 0.9%) by mixing gently. The suspension containing erythrocytes was then centrifuged twice to remove any traces of plasma. Finally, the erythrocyte pellet was re-suspended in 3 mL of normal saline solution to obtain a working solution that was added to 5 mg of each nanocomplex, separately. For the preparation of negative and positive controls, corresponding to 0% and 100% erythrocyte lysis, equal volumes of erythrocyte working suspension were mixed with physiological saline or 1% Triton X-100, respectively. All samples were incubated at 37 °C for 30 min and subsequently centrifuged at 4500 rpm for 10 min to pellet intact red blood cells. The collected supernatants were then left at room temperature for 20 min to promote the conversion of hemoglobin (Hb) to oxyhemoglobin. Absorbance of oxyhemoglobin was measured at 540 nm, and the percentage of erythrocyte hemolysis was calculated using the equation reported below:

$$\mathrm{Hemolysis}\;(\%)=\frac{A_{\mathrm{sample}}-A_{\mathrm{negative}\;\mathrm{control}}}{A_{\mathrm{positive}\;\mathrm{control}}-A_{\mathrm{negative}\;\mathrm{control}}}$$

whereas A_sample_, A_negative control_, and A_positive control_ denote the absorbance of the test sample, negative control, and positive control, respectively.

Hemolytic potential was evaluated in accordance with the following classification: formulations with a hemolysis value of 25% were determined to be at risk for hemolysis.

### 3.6. Preparation of Chitosan-NPs Composite Coating with miR-21

Chitosan-NPs composite coating was prepared by dispersing 50 mg of miR-NPs in 10 mL of deacetylated chitosan from crab shells dissolved to a concentration of 4% (*w*/*v*) in a water solution containing 2% (*v*/*v*) of acetic acid. This solution was used to dip-coat the metallic implant with a 1 min immersion. The miR21-implant was left to dry overnight at room temperature. The coated implant was then treated with a 10 N NaOH solution for 15 min to make the coating-film water insoluble. Finally, it was rinsed several times with phosphate-buffered solution (PBS) until its pH was neutralized. The structure of the implant was analyzed in detail by scanning electron microscopy (SEM). Specimens were sputter coated with AuPd before being observed at 20 kV by the FEI Quanta 200 FEG scanning electron microscope.

### 3.7. In Vitro Cell Models

Human periodontal ligament fibroblasts (hPDLFs) and primary human umbilical vein endothelial cells (HUVECs) were obtained from Thermo Fisher Scientific (Rome, Italy). hPDLFs were maintained in Dulbecco’s Modified Eagle Medium (DMEM), while HUVECs were cultured in RPMI-1640–based medium. The hPDLF culture medium was supplemented with 10% fetal bovine serum (FBS), penicillin (100 U/mL), and streptomycin (100 µg/mL). HUVECs were grown in phenol red–free medium containing 2% serum. All cell cultures were incubated at 37 °C in a humidified environment with 5% CO_2_. Routine quality control assays were performed to exclude microbial contamination, including Mycoplasma testing. Experimental procedures were initiated when cells reached approximately 80% confluence. To induce an inflammatory periodontal-like condition, cells were exposed to interleukin-1β and lipopolysaccharide (IL-1β/LPS, 100 ng/mL). For osteogenic differentiation, hPDLFs were cultured in osteogenic induction medium (OIM) consisting of α-MEM supplemented with 15% FBS, 2 mM L-glutamine, 10 mM sodium β-glycerophosphate, 100 µM L-ascorbic acid-2-phosphate, penicillin G (100 U/mL), streptomycin (100 µg/mL), and fungizone (0.25 µg/mL). Cells were harvested after 21 days of cultivation to assess osteogenic differentiation and mineralization. For miR-21 release by titanium implant were used cell culture inserts (Millicell^®^ 24 well; Sigma-Aldrich, St. Louis, MO, USA). Differences between culturing conditions were assessed by measuring ALP activity and calcium concentration, Alizarin red staining, qPCR analysis.

### 3.8. Cell Viability and Proliferation

Cytocompatibility of miR21-NPs and miR21-implant was assessed for 72 h using the Cell Counting Kit-8 assay (CCK-8, Dojindo Molecular Technologies, Kumamoto, Japan) as reported by Valentino et al. [59]. The results were expressed as a percentage relative to the control and calculated by the equation:

$$\mathrm{Cytocompatability}\;(\%)=\frac{{\mathrm{OD}}_{\mathrm{sample}}}{{\mathrm{OD}}_{\mathrm{control}}}\;\times 100$$

where OD_sample_ was the optical density of treated cells with NPs complexes, OD_control_ was the optical density of the untreated cells.

### 3.9. Cellular Uptake

Cellular internalization of miR-21 was evaluated by fluorescence microscopy. Human periodontal ligament fibroblasts (hPDLFs) were seeded onto microscope slides placed in 24-well plates containing cell culture inserts at a density of 4.5 × 10^4^ cells per well. Cells were exposed to fluorescently labeled miR-21-loaded nanoparticles (miR21-NPs) for 24 h at 37 °C. Following incubation, the inserts were removed and cells were washed five times with ice-cold phosphate-buffered saline (PBS) to remove non-internalized nanoparticles. Cells were subsequently fixed with 3.7% paraformaldehyde for 15 min. Slides were mounted with coverslips using ProLong Gold Antifade mounting medium containing DAPI (Thermo Fisher Scientific, Milan, Italy). Fluorescence images were acquired using a Cytation 3 Cell Imaging Multi-Mode Reader (Biotek, Agilent, Milan, Italy).

### 3.10. miR-21 Transfection and Osteogenic Activity Assay

hPDLF cells (1 × 10^5^) were seeded under standard conditions into a 6-well plate containing free antibiotics DMEM and were transfected for 24 h with miR21-NPs complexes. Lipofectamine RNAiMAX was used as a positive control. Total RNA was extracted from cells using Trizol reagent (Qiagen, Milan, Italy). To verify miR-21 transfection, TaqMan retro-transcription assay was used (Thermo-Fisher Scientific, Milan, Italy). For retro-transcription, total RNA (0.2 μg) was treated as described in Calarco et al. [60] and amplified by qPCR using specific primers for RUNX2, OCN, ALP, OPN, and GAPDH, as listed in Table 1. Quantitative PCR (qPCR) was run on a 7900HT Fast Real time-PCR System (Thermo-Fisher Scientific, Milan, Italy). The reactions were performed using TaqMan and SYBR Green PCR Master mix (Applied Biosystems, Warrington, UK). All reactions were performed in triplicate and normalized to the housekeeping genes RNAU6 and GAPDH. Relative gene expression was calculated using the 2^−ΔΔCt^ method, and results are reported as mean ± SD. *Alkaline Phosphatase (ALP) Activity.* On day 7, ALP activity was measured using an Alkaline Phosphatase Assay Kit (Sigma-Aldrich, Milan, Italy) following the manufacturer’s instructions. Cellular ALP levels were normalized to total protein content. *Alizarin Red S (ARS) Staining.* On day 14, mineralized nodules in hPDLFs cultured under different conditions were visualized using ARS staining. Cells were washed with PBS, fixed in 4% paraformaldehyde for 30 min, then stained with 2% ARS solution for 5–10 min. Excess stain was removed by washing with PBS.

### 3.11. Scratch Migration Assay

Approximately 1 × 10^5^ hPDLF cells were plated in 24-well plates containing a cell culture insert. Following overnight incubation, cells were transfected with either a miR21-implant or miR-NC. Scratch wounds were generated in confluent monolayers using a 200 μL pipette tip. Cells were then rinsed multiple times with culture medium to remove detached cells and debris. Wound closure was assessed after 24 h by measuring the gap width relative to the initial wound at 0 h.

The migrating cells were fixed with 3.7% paraformaldehyde. The images of the migrating cells were obtained using Cytation 3 Cell Imaging Multi-Mode Reader (Agilent Technologies, Santa Clara, CA, USA). The number of migrating cells was counted with ImageJ software (National Institute of Health, Wayne Rasband, MD, USA). Each experiment was conducted in triplicate.

### 3.12. Tube Formation Assay

To evaluate the tubulogenic activity of HUVECs under conditioned media (miR21-NP_20_), the formation of capillary-like structures was examined using a growth-factor-reduced extracellular matrix (MatrigelTM; Corning Incorporated, Corning, NY, USA). The experiment was conducted as reported by Valentino et al. [59].

### 3.13. Statistical Analysis

All values were expressed as mean ± standard deviation (SD). Each experiment was performed at least three times. A one-way analysis of variance (ANOVA) was used for statistical analysis, followed by Bonferroni’s test for multiple comparisons to determined significance differences between groups. All the data were analyzed with the GraphPad Prism version 8.01 statistical software package (GraphPad, San Diego, CA, USA).

## 4. Conclusions

This study demonstrates that lecithin-based cationic nanoparticles (LEC-PEI NPs), prepared via aminolysis of lecithin with low-molecular-weight PEI, serve as highly efficient non-viral vectors for the delivery of miR-21 in hPDLFs. The engineered nanoparticles exhibited high miR-21 loading capacity, excellent stability, and negligible cytotoxicity even after 72 h of exposure. Optimization of the N/P ratio, along with the uniform dispersion of miR21-NPs in a chitosan solution, effectively protected miR-21 from RNase-mediated degradation. Compared to the widely used Lipofectamine RNAiMAX, the nanosized LEC-PEI system achieved approximately 50% higher transfection efficiency, ensuring robust intracellular delivery. When embedded in a chitosan coating on titanium implants, miR21-NPs provided a controlled and sustained release profile, with the polymer coating acting as a coadjuvant to modulate long-term administration. Functionally, miR-21 delivery restores osteogenic and differentiation markers such as Runx2, ALP, and OCN, and significantly enhances cell proliferation, migration, and osteogenic gene expression under inflammatory conditions. Collectively, these effects contribute to improved peri-implant tissue regeneration, prevention of bone loss, and promotion of stable osseointegration. Then, the application of miR21-implant serves as a multifunctional, biocompatible, and targeted strategy for preventing peri-implantitis. The encouraging in vitro results provide a strong rationale for further evaluation of in vivo models to validate the therapeutic potential and long-term efficacy of this gene-eluting dental implant system.

## Figures and Tables

**Table 1:** Primers used for qRT-PCR.

Gene	Accession Number	Forward (5′–3′)	Reverse (5′–3′)
*RUNX2*	NM_001024630	ACCGTCTTCACAAATCCTCCC	CTGTCTGTGCCTTCTGGGTT
*OCN*	NM_199173.6	CCACCGAGACACCATGAGAG	CCATAGGGCTGGGAGGTCAG
*ALP*	NM_001632.5	GGGCAACTTCCAGACCATTG	GCTTTCTTGGCCCGATTCAT
*OPN*	NM_001200.4	TGTATCGCAGGCACTCAGGTCA	CCACTCGTTTCTGGTAGTTCTTC
*GAPDH*	NM_001256799.3	TCGTGGAAGGACTCATGACC	ATGATGTTCTGGAGAGCCCC

## Data Availability

The original contributions presented in this study are included in the article/Supplementary Materials. Further inquiries can be directed to the corresponding authors.

## Supplementary Materials

The following supporting information can be downloaded at: https://www.mdpi.com/article/10.3390/pharmaceutics18010142/s1, Figure S1: Cytotoxicity of LEC-PEI based NPs, miR21-NPs and miR21-implant; Table S1: Effect of lecithin/water ratio, sonication time, and amplitude on nanoparticle size and polydispersity index (PDI).
