# Supplementary material for: Innovative Titanium Implants Coated with miR-21-Loaded Nanoparticle for Peri-Implantitis Prevention
Source: Pharmaceutics. 2026 Jan 22;18(1):142. doi: 10.3390/pharmaceutics18010142 (PMC12845439; doi:10.3390/pharmaceutics18010142)
Supplement: Supplementary file 1 [file pharmaceutics-18-00142-s001.zip › pharmaceutics-4067737-supplementary.pdf]

pharmaceutics

MDPI

Supplementary Materials: Innovative Titanium Implants Coated with miR-21-Loaded Nanoparticle for Peri-Implantitis Prevention

Anna Valentino, Raffaele Conte, Pierfrancesco Cerruti, Roberta Condò, Gianfranco Peluso and Anna Calarco

Table S1. Effect of lecithin/water ratio, sonication time, and amplitude on nanoparticle size and polydispersity index (PDI).

Code	Coconut oil/Water Ratio (O/W)	Sonication Time	Sonication Amplitude (%)	Size (nm) ± SD	PDI ± SD
AA	1:1	2 min	60	152.4 ± 5.3	0.034
AB	1:1	4 min	60	150.9 ± 4.8	0.033
AC	1:1	8 min	60	149.8 ± 5.1	0.034
AD	1:1	2 min	83	151.6 ± 4.9	0.033
AE	1:1	4 min	83	150.7 ± 4.2	0.032
AF	1:1	8 min	83	149.5 ± 5.0	0.034
BA	1:2	2 min	60	137.2 ± 3.6	0.027
BB	1:2	4 min	60	136.5 ± 3.4	0.026
BC	1:2	8 min	60	135.9 ± 3.8	0.027
BD	1:2	2 min	83	136.8 ± 3.3	0.028
BE	1:2	4 min	83	136.2 ± 3.0	0.026
BF	1:2	8 min	83	135.5 ± 3.5	0.027
CA	1:4	2 min	60	105.1 ± 4.0	0.018
CB	1:4	4 min	60	104.7 ± 9.6	0.019
CC	1:4	8 min	60	103.9 ± 11.2	0.019
CD	1:4	2 min	83	111.2 ± 3.9	0.018
CE	1:4	4 min	83	110.9 ± 3.5	0.017
CF	1:4	8 min	83	110.5 ± 3.2	0.017
DA	1:8	2 min	60	110.8 ± 2.9	0.022
DB	1:8	4 min	60	109.7 ± 3.4	0.023
DC	1:8	8 min	60	108.9 ± 3.7	0.024
DD	1:8	2 min	83	110.3 ± 2.7	0.022
DE	1:8	4 min	83	109.5 ± 3.2	0.022
DF	1:8	8 min	83	108.7 ± 3.6	0.023

 https://doi.org/10.3390/pharmaceutics18010142

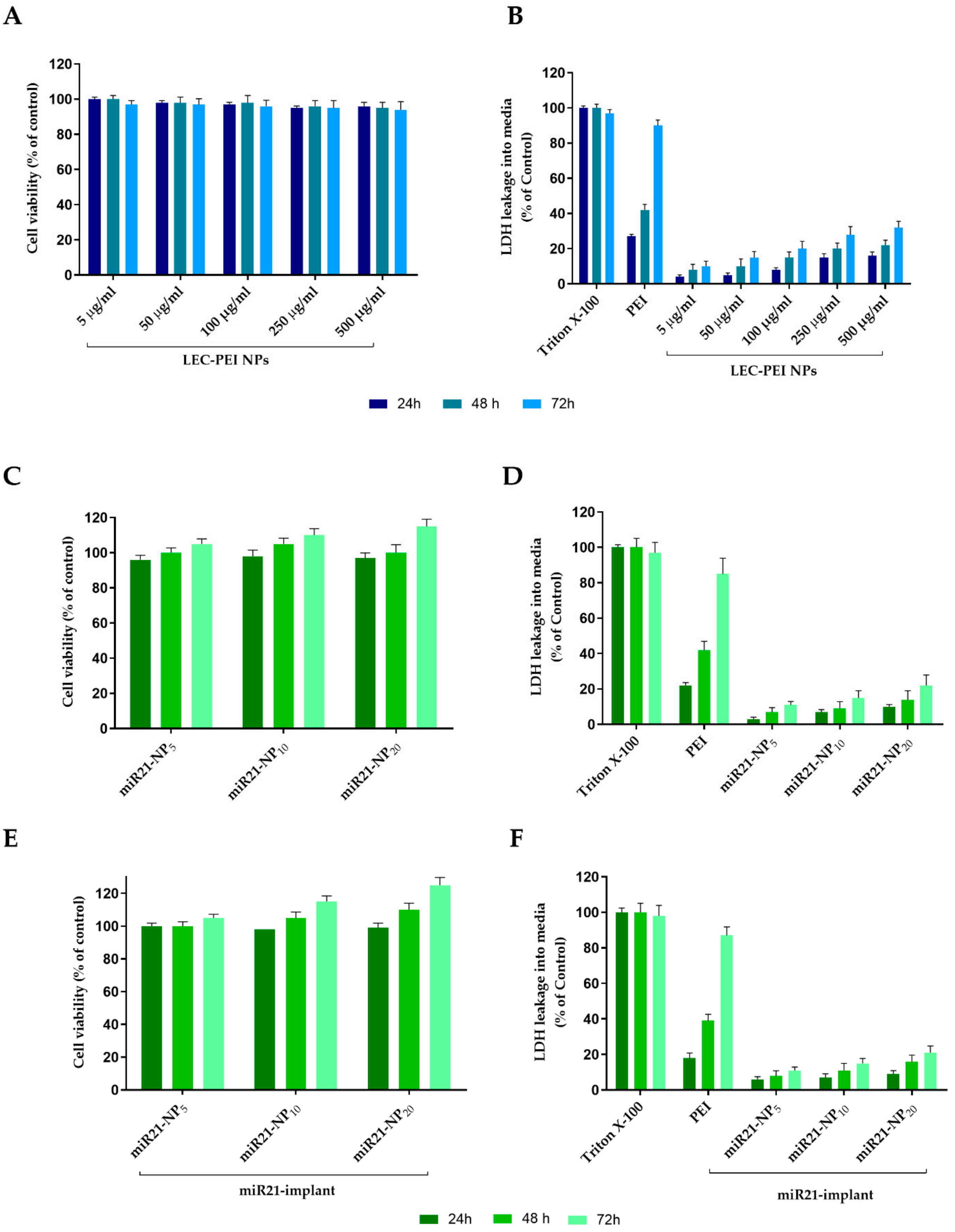

Figure S1. Cytotoxicity of LEC-PEI based NPs, miR21-NPs and miR21-implant. Cytotoxicity was determined in hPDLF cells after 24, 48, and 72 h of incubation with varying concentrations of LEC-PEI NPs, miR21-NPs and miR21-implant using: (A, C, and E) Cell Counting Kit- 8 (CCK-8) assay and, (B, D, and F) the Lactate Dehydrogenase (LDH) assay. Untreated cells and Triton X-100 were used as control. Data are presented as the mean ± SD for three independent measurements.

https://doi.org/10.3390/pharmaceutics18010142
